# Antitumor efficacy of Kisspeptin in human malignant mesothelioma cells

**DOI:** 10.18632/oncotarget.25018

**Published:** 2018-04-10

**Authors:** Vincenza Ciaramella, Carminia Maria Della Corte, Concetta Di Mauro, Stefano Tomassi, Salvatore Di Maro, Teresa Troiani, Erika Martinelli, Roberto Bianco, Sandro Cosconati, Riccardo Pierantoni, Rosaria Meccariello, Rosanna Chianese, Fortunato Ciardiello, Floriana Morgillo

**Affiliations:** ^1^ Oncologia Medica, Dipartimento di Internistica Clinica e Sperimentale “F. Magrassi”, Università degli Studi della Campania Luigi Vanvitelli, Naples, Italy; ^2^ Oncologia Medica, Dipartimento di Medicina Clinica e Chirurgia, Università degli Studi di Napoli “Federico II”, Naples, Italy; ^3^ DISTABIF, Università degli Studi della Campania Luigi Vanvitelli, Caserta, Italy; ^4^ Dipartimento di Medicina Sperimentale sez ‘F. Bottazzi’, Università degli Studi della Campania Luigi Vanvitelli, Naples, Italy; ^5^ Dipartimento di Scienze Motorie e del Benessere, Università di Napoli Parthenope, Napoli, Italy

**Keywords:** mesothelioma, KiSS1, biomarker, metastasis, EMT

## Abstract

**Purpose:**

Kisspeptin signaling, *via* its receptors GPR54, could be an essential players in the inhibition of mesothelioma progression, invasion and metastasis formation. The loss of KiSS1 by tumor cells has been associated with a metastatic phenotype but the mechanistic insights of this process are still unknown.

**Experimental design:**

The blockade of the metastatic process at early stage is a hot topic in cancer research. We studied the role of KiSS1 on proliferation, invasiveness, migration abilities of mesothelioma cell lines focusing on the effect on epithelial-to-mesenchymal transition (EMT).

**Results:**

Treatment with the KiSS1 peptide or with a synthesis peptide with longer half-life, the FTM080, significantly inhibited cell proliferation, migration and invasion of mesothelioma cell lines; the same treatment reduced the activity of MMP-2 and MMP-9 determining consequently a marked reduction in the invasiveness of primary tumors and metastases. Thespecificexpression of EMT markers, as E-caderin, Vimentin, Slug and Snail, suggested the inhibition of EMT after treatment with KiSS1 as well as the preservation of epithelial components.

**Conclusion:**

Our results support anti-proliferative effect of KiSS1 in cancer cells and suggest that targeting the KiSS1/GPR54 system may represent a novel therapeutic approach for mesothelioma.

## INTRODUCTION

One of the rarest but most aggressive tumors that is growing in the worldwide, is the malignant mesothelioma (MM). MM arises often from the mesothelial cells surrounding pleura, peritoneum and, occasionally, the pericardium. MM occurs between 50 and 70 years of age more frequently in males with a male/female ratio 5:1 [1, 2, 3] and it is commonly attributed to occupational exposures to asbestos, a group of fibrous silicates used as needle-like amphiboles or curly serpentine fibers. The nature and bio-persistence of these inhaled fibers may be key to carcinogenic events that occur during the long latency periods (30-45 years) [4]. MM is classified into 3 major histological subtype: epithelioid, sarcomatoid and biphasic (desmoplastic, fibrotic and many others). The epithelioid subtype is the most common and has the best prognosis [5]. The therapeutic approach used for MM consists mostly of combination cisplatin and pemetrexed chemotherapy and mesothelin, glycoprotein normally expressed on the surface of mesothelial cells, remains to date the only marker used in the diagnosis of MM [6]. As in other solid tumors, the pathogenesis of MM occurs gradually through the acquisition of some typical characteristics of malignant cells such as suppression of apoptosis, unlimited capacity for cell replication, tissue invasion and metastasis formation.

The set of events, both morphological and functional, that transform adherent epithelial cells in motile cells able to migrate and invade the extracellular matrix is defined as epithelial-to-mesenchymal transition (EMT). At the cellular level, the EMT mechanism is regulated by specific molecular signals: epithelial cells lose the expression of component of epithelial cell junction, such as E-cadherin, and increase in expression of Vimentin, Slug and Snail that are currently used to identify cells that have undergone an EMT and have been isolated in circulating tumor cells [7]. Cancer cells adopt EMT process in the switch of early stage tumors into dedifferentiated and more malignant states [8, 9]. Increasing evidence indicates that aberrant activation of the developmental EMT program contributes to tumor invasion, metastatic dissemination and acquisition of therapeutic resistance [10]. In this contest, the role of EMT in mesothelioma is largely unknown and cancer research is focusing on the identification of molecules involving in anti-metastatic activity.

Among metastasis-suppressor genes, KiSS1 and its only G-protein coupled receptor, GPR54, seems to act late in the metastatic cascade by preventing growth of the metastatic cells [11]. The KiSS1 gene mapped to chromosome 1q32 and encodes for a 145 amino acid protein which, following proteolytic cleavage, generates a family of Kisspeptins (Kps), including Kp-10, -13, -14 and -54 [12]. KiSS1 was first isolated in melanoma cells [13, 14] by identifying it as a suppressor sequence (SS); the letters “Ki” were added to the prefix “SS” to form “KiSS” in homage to the location of its discovery, Hershley (Pennsylvania), home of the famous “Hershley Chocolate Kisses” [12]. The expression profile and the mechanism of action in tumor metastasis of Kps and GPR54 is still unclear. It has been reported that down regulation of KiSS1 was associated with more aggressive ovarian cancer [15] and caused tumor growth and invasion becoming a strong prognostic factor in gastric carcinoma patients [16]. Moreover, KiSS1 may represent a crucial factor in cancer patient survival in term of overall and disease-free survival: low expression of this peptide determined vascular invasion in urinary bladder carcinoma [17] while its high expression level inhibited cellular proliferation through intracellular Ca^++^ release [18]. The purpose of this work is to study the role of KiSS1 in mesothelioma and to define the pathway involved in cancer progression or survival.

## RESULTS

### Effect of Kisspeptin on the invasiveness of mesothelioma cell lines: proliferation, migration, invasion and colony forming assays

**Figure 1:**
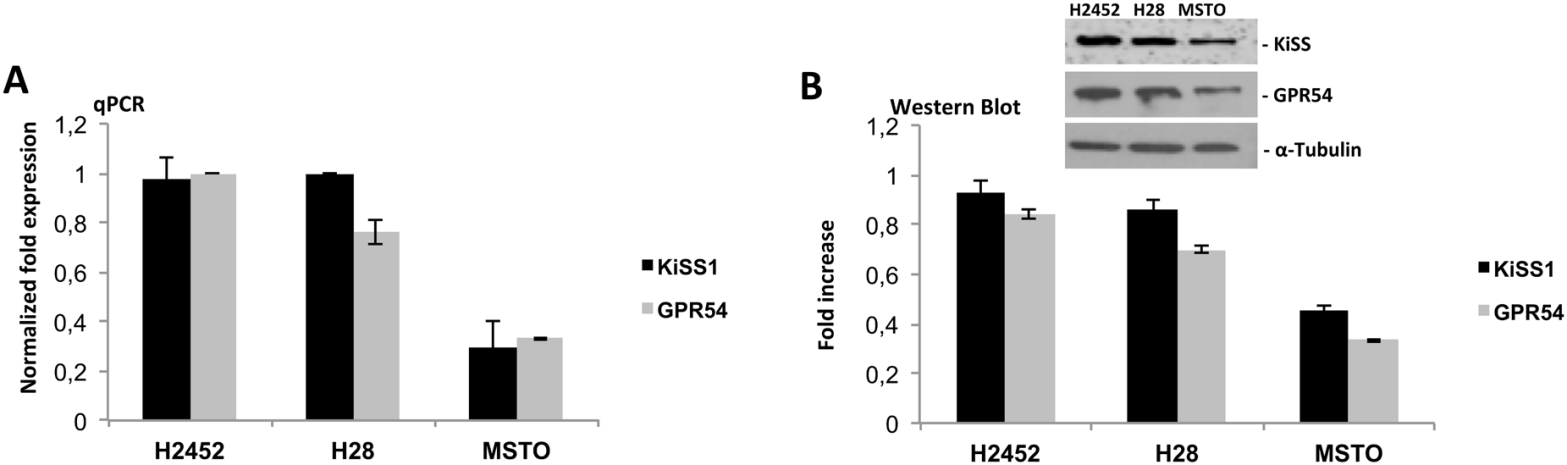
Expression analysis of KiSS1/GPR54 system in mesothelioma cell lines **(A)** qPCR and **(B)** Western blot analysis on total mRNA and protein of KiSS1 and GPR54 from mesothelioma cell lines: H2452, H28 and MSTO. Gene and protein expression levels was determined by normalizing to GAPDH and α-Tubulin, for qPCR and Western Blot respectively.

To study the role of Kisspeptin and its G-protein coupled receptor GPR54 in mesothelioma, we first evaluated the expression level of KiSS1 and GPR54 in human mesothelioma cell lines. We selected for our experiment three cell lines, representative of different mesothelioma subtypes: H2452, epithelial mesothelioma, H28, sarcomatoid mesothelioma, and MSTO, biphasic mesothelioma. Quantitative expression of KiSS1 and GPR54 was detected by qPCR and Western Blot analysis on total RNA and protein from mesothelioma cell lines (Figure 1A, 1B), respectively. The analysis revealed the presence of both transcripts and proteins in all cell lines used; higher expression levels were observed in H2452 and H28 cell lines than in MSTO cell line were the expression appeared significantly lower.

**Figure 2:**
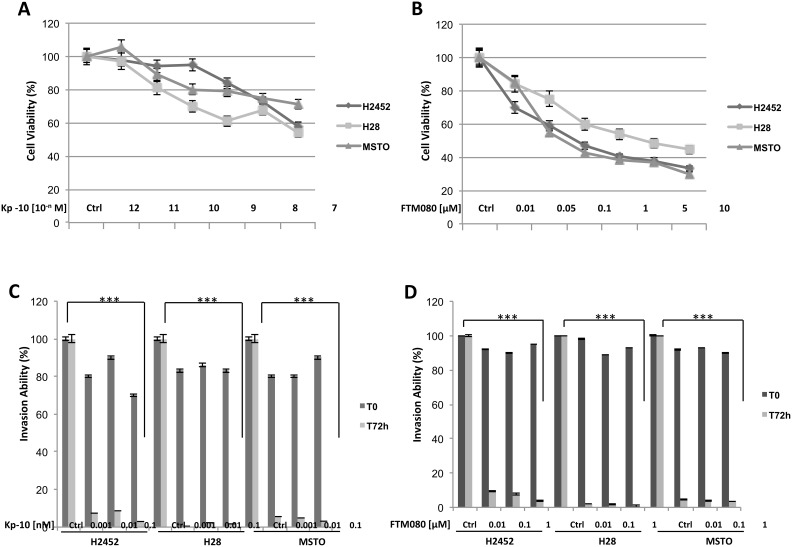
Effect of Kisspeptin and FTM080 on the proliferation and the invasiveness of mesothelioma cell lines **(A-B)** Cell viability, **(C-D)** invasion ability. Data are the average ± SD of three independent experiments, each performed in triplicate. Asterisks indicate statistical significance, as determined by the Student-t test (^***^ *P* ≤ 0.001).

**Figure 3:**
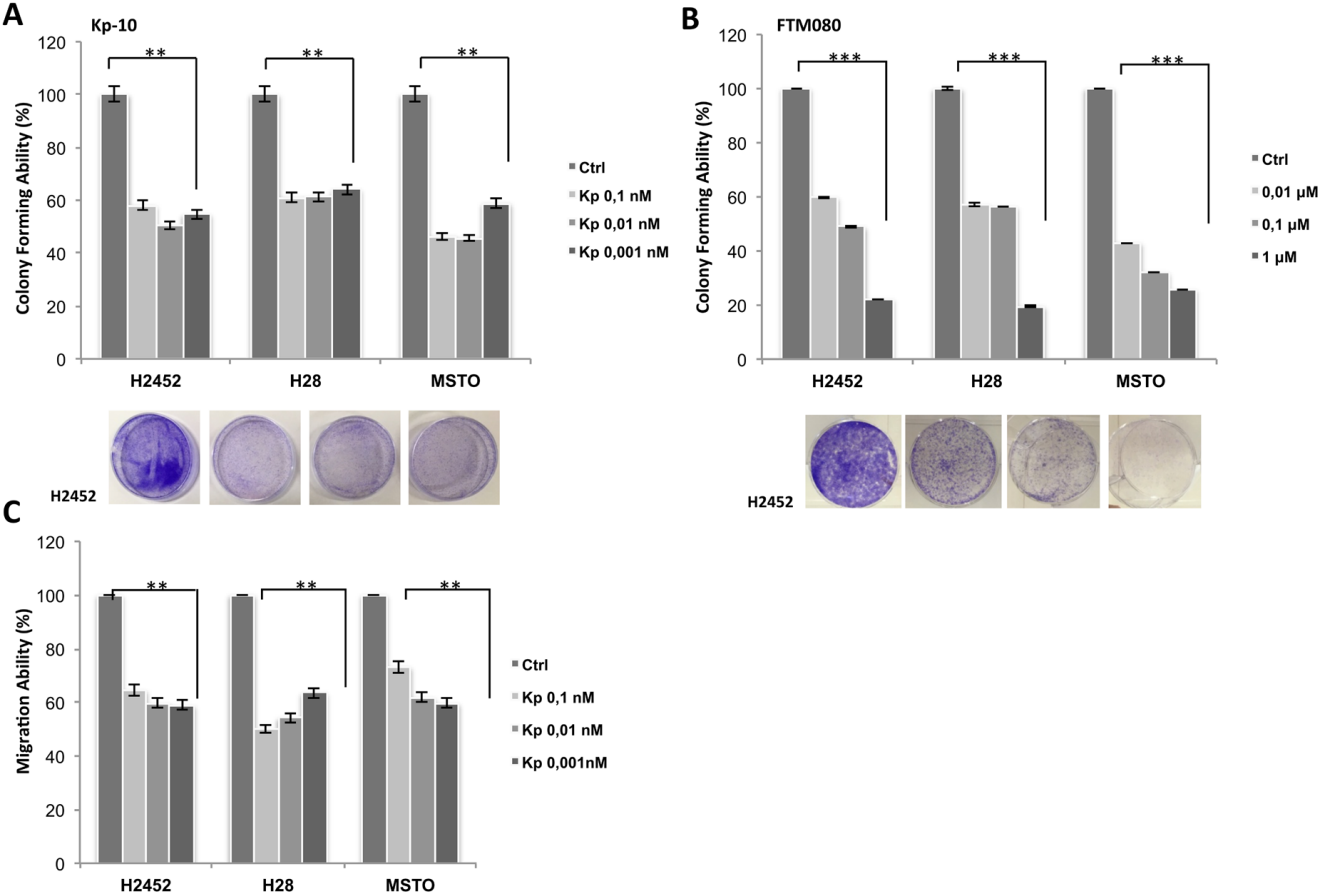
Effect of Kisspeptin and FTM080 on the migration and colonies formation of mesothelioma cell lines **(A-B)** Colony formation assay, **(C)** migration ability. Data are the average ± SD of three independent experiments, each performed in triplicate. Asterisks indicate statistical significance, as determined by the Student-t test (^**^ *P* ≤ 0.01; ^***^ *P* ≤ 0.001).

One of the main characteristics of malignant cells is their ability to growth in monolayers and in colonies, to invade and migrate. As shown in Figure 2A, a dose dependent inhibition of cell viability after kisspeptin-10 (Kp-10) treatment was observed in H2452, H28 and MSTO human mesothelioma cell lines: cells were initially cultured in the presence of increasing doses of Kp-10 from 10^-12^ to 10^-7^ M and analyzed after 72 hours, observing a progressive decrease in cell proliferation with higher doses, with IC50 value between 10^-9^ and10^-7^ M (Figure 2A). Therefore, we evaluated the abilities of these cells to invade, migrate and to form colonies *in vitro* (Figure 2C, 3A, 3C). Following Kp-10 treatment at the range dose 0.001, 0.01, 0.1 nM, cells seemed to loose their aggressiveness and probably their high metastatic abilities; in fact our results displayed a strongly reduction to the > 80% of cell invasion in presence of Kp-10 (Figure 2C) than the control cells without Kp-10 treatment. Similar data were obtained for cell migration and anchorage-independent colony forming abilities. In particular, with a dose of Kp-10 0.1 nM migration ability of mesothelioma cells decreased to about 50% in H2452 cells and about 60% in H28 and MSTO if compared to untreated controls (Figure 3A, 3C). Since Kp-10 has a rather short half-life, we have chosen to carry out the same *in vitro* experiments using a new synthesis peptide with longer half-life, the FTM080, a Kisspeptin receptor agonist synthesized based on the known sequence of Kp-10 to verify its effect on mesothelioma cell lines used. Surprisingly we obtained a stronger effect in terms of inhibition of proliferation (Figure 2B), invasion (Figure 2D) and formation of colonies after treatment with FTM080 (Figure 3B) demonstrating that the anti-metastatic effect could be enhanced using a synthetic molecule.

### Effect of Kisspeptin on intracellular signaling in mesothelioma cell lines: Epcam, Zymography and protein analysis

**Figure 4:**
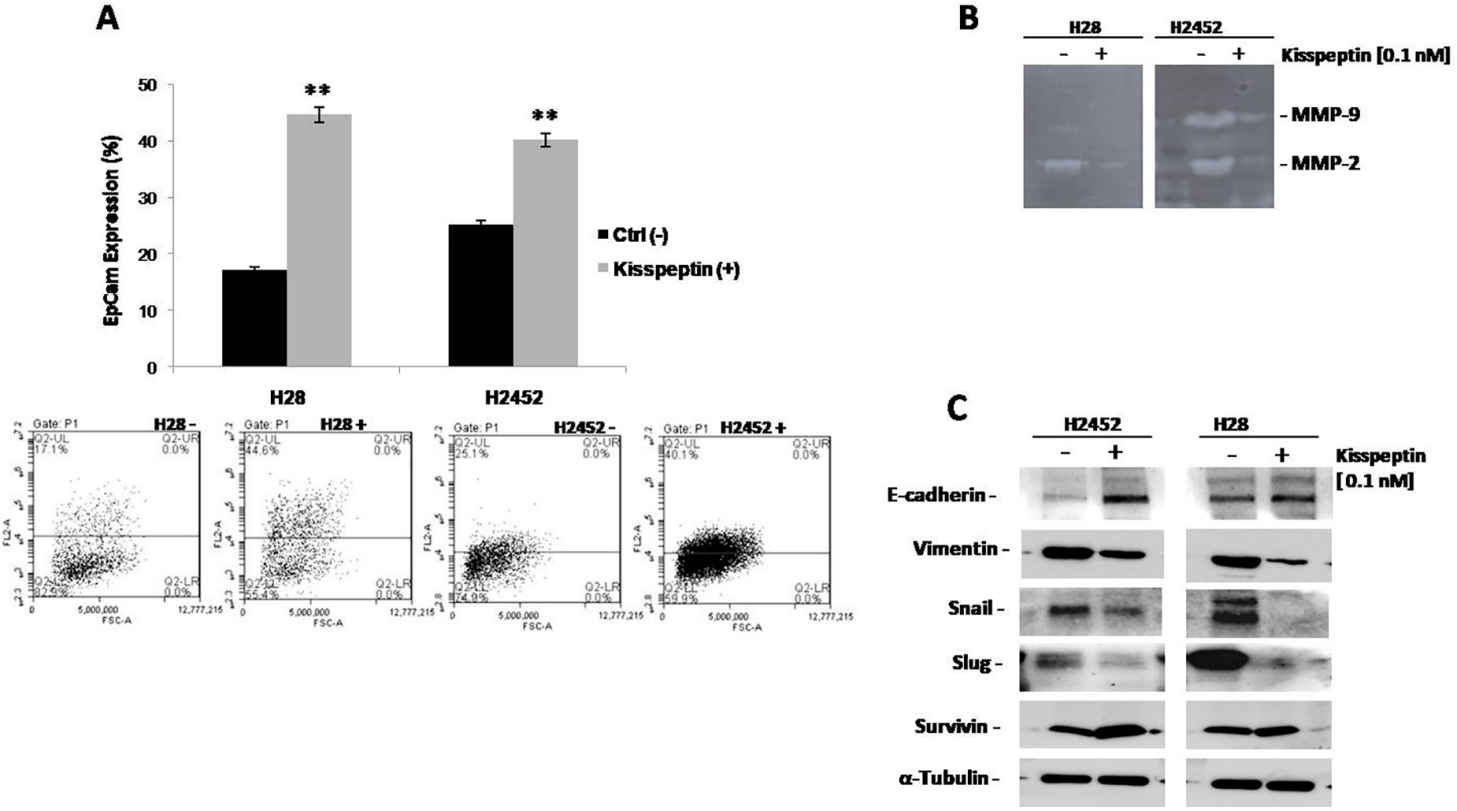
Effect of Kisspeptin on intracellular signaling in mesothelioma cell lines: Epcam, Zymography, Western Blot **(A)** Flow cytometry analysis with EpCAM staining in H28 and H2452 mesothelioma cell lines after treatment with Kp-10. Asterisks indicate statistical significance, as determined by the Student-t test (^**^ *P* ≤ 0.01). **(B)** MMP-2 and MMP-9 activities determined by gelatin zymography in the conditioned media of H28 and H2452 mesothelioma cell lines before and after treatment with Kp-10. **(C)** Western blot analysis for EMT-related protein E-cadherin, Vimentin, Snail, Slug and Survivin were performed on protein lysates from both H28 and H2452 mesothelioma cell lines following treatment with the indicate concentration of Kp-10. α-Tubulin was included as a loading control.

We next examined whether mesothelioma cell lines show evidence of molecular changes known to occur during the epithelial-to-mesenchymal transition (EMT). It has been demonstrated that cancer cells undergo EMT during tumor progression and metastasis, in which they lose numerous epithelial characteristics and acquire invasive properties and stem features [9]. We selected H28 and H2452 cells, to investigate the function of Kisspeptin in the phenotypic change that take place in malignant cell and in the formation of metastases. Firstly, we quantified by flow cytometry analysis the epithelial marker, Ep-Cam, in mesothelioma cells after Kp-10 treatment; H28 and H2452 cells were treated with 0.1 nM Kp-10 for 48 hours and marked with Ep-Cam antibody which functions as an epithelial cell adhesion molecule (Figure 4A). The analysis revealed a strong over expression of the epithelial element: an Ep-Cam increase of 27% and 15%, in H28 and H2452 respectively, was observed following Kp-10 treatment than the control cells, indicating that the presence of Kisspeptin is associated with epithelial phenotype and thus favors a less aggressive behavior than the more aggressive mesenchymal one.

EMT also causes the reorganization of the extracellular matrix (ECM) as many EMT-inducing factors up-regulate the expression of ECM molecules such as matrix metalloproteases (MMPs). In this contest, MMP-2 and MMP-9, play a critical role in cancer cell migration and invasion by stimulating the degradation of ECM and their increased expression is associated with disease progression. H28 and H2452 mesothelioma cells, exhibit considerable reduction in MMP-2 and MMP-9 after 0.1 nM Kp-10 treatment as highlighted by gelatin zymography analysis in Figure 4B. The reduction in protein production corresponded to a reduced activity of MMP-2 and MMP-9, and demonstrated the “protective function” of Kisspeptin in term of cell change architecture and behavior associated to tumor recurrence.

To approach the molecular pathway regulated by Kisspeptin, we studied the typical markers of the EMT program during development and tumor invasion. Western blot analyses were done on protein extracts from H28 and H2452 cells that were treated with 0.1 nM Kp-10 for 48 hours. We observed the up regulation of E-cadherin, adhesion molecule essential for the formation and maintenance of the epithelial cell phenotype. On the contrary, we revealed a consistently loss of mesenchymal markers such as Vimentin, Snail and Slug suggesting that these cells, under the effect of Kisspeptin, retain the epithelial phenotype by inhibiting the EMT. Moreover, we observed an increase in Survivin expression that let to hypothesize, a pro-survival function on cells after treatment (Figure 4C).

### *In vivo* effects of the treatment with Kisspeptin agonist

As our *in vitro* studies revealed changes in the expression of mesenchymal proteins, Snail and Vimentin, and in the activity of MMP-2 and MMP-9 on cancer cells treated with Kp-10 (Figure 4), we also investigated whether the Kisspeptin treatment blocks tumor metastatic behavior *in vivo*. Several studies have shown that Kp-10 is completely degraded in murine serum within 1 hour; in contrast FTM080, the pentapeptide agonist that exhibits equipotent GPR54 activation to Kp-10, has a half-life in murine serum of 6.6 hours [25] and offers more stability under physiological conditions. Therefore we performed an artificial metastasis assay by injecting H2452 cells into the tail vein of Balb/c nude mice (six mice per control group and six mice per treatment group), and treating them with FTM080 (10 mg/kg p.o. four days a week, for three weeks). At the end of treatment period, mice were sacrificed and organs (lungs, liver and spleen) were analyzed macroscopically to search tumoral lesions. All mice, both in control and treatment group, showed no macroscopic evidence of lesions (data not shown).

**Figure 5:**
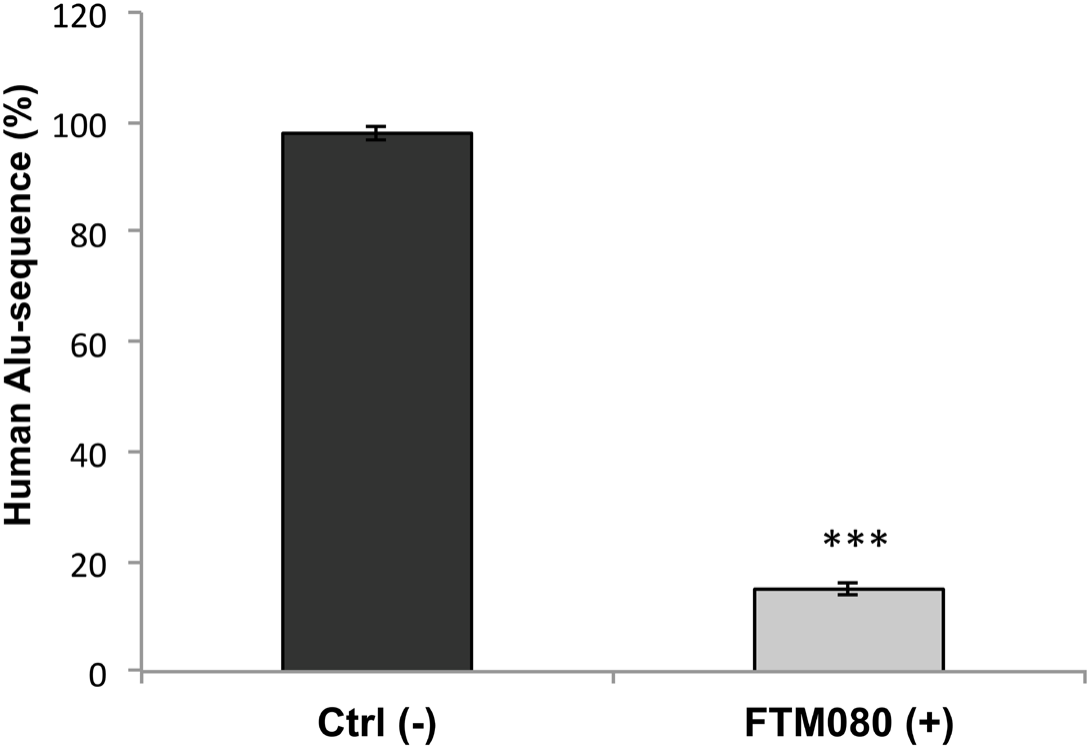
*In vivo* effects of the FTM080 treatment Percentage of human Alu sequences in the lungs of athymic nude mice after tail vein injection with H2452 mesothelioma cells and the indicated treatments. Data are the average ± SD of three independent experiments, each performed in triplicate. Asterisks indicate statistical significance, as determined by the Student-t test (^***^ *P* ≤ 0.001).

A fast and accurate method to evaluate the micrometastasis formation and thus the therapeutic efficacy of anti-metastatic drugs in xenografts is the Alu signal analysis [24]; therefore we measured lung micrometastasis formation quantifying the portion of human DNA in mouse lungs using realtime PCR for human Alu sequences. Untreated mice exhibit a large amount of human DNA expressed as a percentage of human Alu sequences; precisely the amount of Alu portion is about 5 times higher than the mice treated with FTM080, demonstrating the effecacy of *in vivo* anti-metastatic treatment (Figure 5).

## DISCUSSION

Kisspeptins are a group of peptide fragments encoded by the *KiSS1* gene in humans. They bind to Kisspeptin receptor, GPR54, with equal efficacy. Several studies provided convincing evidence of role of this signaling in regulation of cancer development and progression. Although it remains controversial to some degree, a more complete picture of the potential mechanisms behind anti-metastatic signaling emerges from the action of KiSS1 and its receptor, GPR54. KiSS1 has been demonstrated as a suppressor of metastasis in the majority of cancers, including gastric cancer, oesophageal carcinoma, pancreatic, ovarian, bladder and prostate cancer [26]. In this context we have chosen to study the role of Kisspeptin in malignant mesothelioma. First of all, we checked the presence of transcripts and proteins of KiSS1 and its coupled receptor, GPR54, in mesothelioma cell lines H2452, H28 and MSTO observing a prevalence of expression levels in H2452 and H28 lines, epithelial and sarcomatoid mesothelioma, respectively. We subsequently tested *in vitro* the effect of Kisspeptin-10 (Kp-10) on cell proliferation, migration and invasion: the treatment results in a reduction of cell aggressiveness and their high metastatic abilities.

After observing a strong inhibitory effect of Kp-10 on cell migration and invasion, we focused our attention on the EMT mechanism that, by inducing precise molecular signals, leads to a transformation of cells to a more malignant phenotype. In fact, we analyzed epithelial marker Ep-Cam in the mesothelioma cells after treatment with Kp-10, noting a strong expression of this epithelial element in the cells treated with Kp-10 than in untreated control. This evidence suggests that the presence of Kisspeptin favors the maintenance of the epithelial phenotype, that is, a less aggressive behavior of cancerous cells. Therefore, we conducted a protein analysis on mesothelioma cell lines after Kp-10 treatment and we were able to demonstrate that such treatment determines the up-regulation of E-cadherin, a key cell adhesion molecule, and the loss of typical mesenchymal markers such as Vimentin, Snail and Slug suggesting that cells, under the effect of Kp-10, retain the epithelial phenotype by inhibiting the EMT. Probably a pro-survival mechanism is likely to explain the increase in Survivin expression levels in Kp-10 mesothelioma cells.

Besides, a possible explanation for the role played by KiSS1 in cancer biology can be extrapolated from the correlation between KiSS1 and matrix metalloproteases (MMPs), particularly MMP-9 and MMP-2, whose significance in tumor invasion and metastasis formation is well known. Studies on different types of solid tumors such as breast, bladder and pulmonary carcinomas reported that the activation of MMPs, especially MMP-2, is related to the tumor and/or the spread of cancerous cells [27, 28]. Furthermore, it has been shown that MMP-2 production and activation directly correlates with invasion of tumor cells and lymphatic permeation in stomach tumors [29]. In our experiments, performed on H28 and H2452 cell lines, we observed remarkable reductions in MMP-2 and MMP-9 after 0.1 nM of Kp-10 as evidenced by gelatin zymography analysis. Kisspeptin is therefore responsible for a low production and activation of MMPs and thus promotes the retention of cell structure and behavior.

Kispeptin is a peptide with a short half-life, from 30 minutes to a maximum of 1 hour, which makes it quite unstable and difficult to consider as a real drug. For this reason, we synthesized a peptide, FTM080, with a similar sequence but with a half-life of almost 7 hours and we repeated all experiments under the same conditions to test its effectiveness. The results obtained were very significant in terms of inhibiting proliferation, invasion and colonization and allowed us to demonstrate that the use of a synthetic peptide could enhance the anti-metastastic and antineoplastic effect regulated by Kp-10.

In the wake of this discovery, we translated the study of *in vitro* metastatic mechanisms for *in vivo* analysis on nude mice using the agonist FTM080 as a treatment after inoculation with mesothelioma cells. The result obtained was very satisfactory: in fact the analysis performed on the human Alu sequences clearly showed that mice receiving FTM080 had human Alu quantity reduced by more than 5 times the mice they did not receive type of treatment. These data demonstrate that the use of a synthetic agonist molecule of Kp-10 could be used as an effective anti-metastatic treatment and have potential diagnostic and therapeutic applications.

In conclusion, we demonstrated that Kisspeptin could play a key role in the inhibition of metastatic cascade including proliferation, invasion and migration but, also, act directly in intracellular signaling by modifying the activity and expression of specific proteins tightly bound to the epithelial to mesenchymal transition (EMT). Our data suggest that KiSS1/GPR54 system has a “protective” function by inhibiting the transformation of epithelial cells into more aggressive mesenchymal cells. Consequently, our work opens the possibility for using Kisspeptin molecule as therapy in tumors characterized by poor survival, such as mesothelioma.

## MATERIALS AND METHODS

### Cell lines, drugs and chemicals

The human pleural mesothelioma cell lines H2452, H28 and MSTO were provided by American Type Culture Collection (ATCC, Manassas, VA, USA) and maintained in RPMI-1640 (Sigma-Aldrich, Saint Louis, MO, USA) medium supplemented with 10% fetal bovine serum (FBS; Life Technologies, Gaithersburg, MD) and 1% penicillin/streptomycin and were cultured in a humidified atmosphere with 5% CO_2_ at 37°C. The identity of all cell lines was confirmed by STR profiling (Promega, Madison, WV, USA) on an ad hoc basis prior to performing experiments.

Primary antibodies for western blot analysis against E-cadherin, Vimentin, Snail, Slug, Survivin and α-Tubulin were obtained from Cell Signaling Technology; instead, KiSS1 and GPR54 antibodies (sc-18134 and sc-134499, respectively) were obtained from Santa Cruz Biotechnology, Inc. The following secondary antibodies from Bio-Rad were used: goat anti-rabbit IgG, rabbit anti-mouse IgG and monoclonal anti-α-Tubulin antibody (T8203) from Sigma Chemical Co.

Kisspeptin-10 [Human Metastin, 45-54 (H-YNWNSFGLRF-NH_2_)] was purchased from Santa Cruz Biotechnology, dissolved in sterile dimethylsulfoxide (DMSO) and a 2 mM stock solution was prepared and stored in aliquots at −20°C. Working concentrations were diluted in culture medium just before each experiment.

Standard *N*α-Fmoc-protected amino acids, *O*-benzotriazole-*N*, *N*, *N*′, *N*′-tetra-methyl-uroniumhexafluorophosphate (HBTU, purity 99%), *N*, *N*-diisopropylethylamine (DIEA, purity 99%), trifluoroacetic acid (TFA, purity 99%) and piperidine, were purchased from IRIS Biotech (Marktredwitz, Germany). Fmoc-Rink amide-Am resin, triisopropylsilane (TIS) (purity 98 %), 1-hydroxybenzotriazole hydrate (HOBt) (purity > 97 % dry weight, water 12 %), 4-fluorobenzoic acid (purity 98 %) and acetic anhydride (Ac_2_O, purity > 98 %) were purchased from Sigma-Aldrich (Milano, Italy).

### Synthesis of FTM080

Peptides were purified by preparative HPLC (Shimadzu HPLC system) equipped with a C18-bounded preparative RP-HPLC column (PhenomenexKinetex 21.2 mm × 150 mm, 5 μm) and analyzed by analytical HPLC (Shimadzu Prominance HPLC system). Molecular weights of compounds were confirmed by high resolution mass spectrometry using a Thermo Fisher Scientific TSQ Altis Triple Quadrupole LC-MS. A Rink Amide AM-PS resin (312 mg, 0.15 mmol, 0.48 mmol/g) was swelled in a mixture of DCM/DMF 1:1 over 40 min and then washed with DCM (3×1 min) and DMF (3×1 min). Initial resin-bound Fmoc protecting group was removed treating with 20% (v/v) piperidine solution in DMF (1×5 min; 1×25 min). Amide coupling reactions were carried out using 4 equiv (according to the initial loading of the resin) of the Fmoc-protected L-amino acids. To a pre-stirred solution of Fmoc-L-Trp(Boc)−OH (316 mg, 0.60 mmol, 4 equiv) with HBTU (227 mg, 0.60 mmol, 4 equiv) and HOBt (92 mg, 0.60 mmol, 4 equiv) in DMF/DCM 9:1 (4.0 mL), DIPEA (209 μL, 1.20 mmol, 8 equiv) was added and the solution poured to the pre-swollen resin in peptide synthesis reactor. The mixture was gently shaken for 2 hours at room temperature and then washed with DMF (3×1 min) and DCM (3×1 min). Subsequently, to cap the remaining free amines the resin was treated with 3.0 mL of an acetylating solution containing 2 equiv of Ac2O (relative to the initial loading of the resin) and 3 equiv of DIPEA (relative to the initial loading of the resin) in DMF and then shaken for 5 min. Elongation of the linear peptide sequences were obtained by iterative cycles of the aforementioned Fmocdeprotections and coupling reactions with Fmoc-L-amino acids as reported above and according to the amino acidic sequence. Completion of these steps were qualitatively monitored by Kaiser ninhydrine and TNBS [19, 20] test and quantitatively ascertained by Fmoc UV spectroscopic measurements [21]. After last Fmocdeprotection, Phe primary amine was coupled with 4-fluorobenzoic acid (84 mg, 0.6 mmol, 4 equiv) in DMF/DCM 9:1 (4.0 mL), using HBTU (227 mg, 0.60 mmol, 4 equiv) and HOBt (92 mg, 0.60 mmol, 4 equiv) as coupling reagents and DIPEA (209 μL, 1.20 mmol, 8 equiv) as base. The reaction was allowed to stir for 2 hours at room temperature then the resin was washed with DMF (3×1 min) and DCM (3×1 min). Completion of the reaction was qualitatively determined by Kaiser test and TNBS test [19, 20]. The obtained resin-bound peptide was washed with DMF (3×1min), DCM (5×1min), and Et2O (2×1 min) and then dried exhaustively. Peptide cleavage and acidic sensitive protective groups deprotection were simultaneously carried by treatment with a solution of TFA/TIS (95:5, 4 mL) for 3 hours at room temperature. The resin was filtered and the crude peptides precipitated from the TFA solution, diluting to 45 mL with cold Et2O and then centrifuged (6000 rpm × 15 min). The supernatant was carefully removed and the precipitate suspended again in 45 mL Et2O as described above. The resulting wet solid was dried for 1 hours under reduced pressure, redissolved in water/acetonitrile (9:1) and purified by reverse-phase HPLC (solvent A: water + 0.1 % TFA; solvent B: acetonitrile + 0.1 % TFA; from 10 to 90% of solvent B over 25 min, flow rate: 10 mL min^−1^) unless otherwise stated. Fractions of interest were evaporated from organic solvents under reduced pressure, freezed and then lyophilized. The obtained product was characterized by analytical HPLC (solvent A: water + 0.1 % TFA; solvent B: acetonitrile + 0.1 % TFA; from 10 to 90% of solvent B over 20 min, flow rate: 1 mL min^−1^, unless otherwise stated) and High Resolution Mass Spectrometry. FTM080: 98 mg, overall yield: 82 %, purity: ≥ 95%, tR 20.55 min, HRMS (ESI-MS): Calculated: 799,40553 for C41H52FN10O6 [M+H]+, found: 799.40516.

### Expression analysis: mRNA and protein

#### Quantitative real time PCR (qPCR)

Cells total RNA was extracted using Trizol reagent (Life Technologies). Reverse transcriptase reaction was carried out to convert 1μg of isolated RNA into cDNA using Super-Script reverse transcriptase III (Life Technologies) according to the manufacturer instruction. Expression levels of genes encoding for KiSS1 and GPR54 were analyzed using qPCR. The primers used were: KiSS1: 5’-atgaactcactggtttcttgg-3’ and 5’-ccccacagaggccacctttt; GPR54: 5’-gcagaccgtcaccaatttct-3’ and 5’-gggaacacagrcacgtacca-3’; GAPDH: 5’-agatgacccagatcatgtttgaga-3’ and 5’-accagaggcatacagggacaa-3’. Amplifications were done using the SSo Fast EvaGreen supermix (Bio-Rad). All samples were run in duplicate, using the Mastercycler CFX-96 (Bio-Rad) and relative expression of genes was determined by normalizing to GAPDH, used as internal control gene. All assays included a melting curve analysis for which all samples displayed single peaks for each primer pair. To calculate the fold change in value it was used the 2- ΔΔCt method. Nonspecific signals caused by primer dimers were excluded by dissociation curve analysis and use of non-template controls. Data were then reported as mean fold change ± SD over the minimal value arbitrarily assigned to a reference sample and ANOVA followed by Duncan’s test for multigroup comparison was carried out to assess the significance of differences.

#### Protein expression analysis

Protein lysates, derived from mesothelioma cells following treatment, were obtained by homogenization in RIPA lyses buffer [0.1% sodium dodecylsulfate (SDS), 0,5% deoxycholate, 1% Nonidet, 100 mmol/L NaCl, 10 mmol/L Tris–HCl (pH 7.4), 0.5 mmol/L dithiotritol, and 0.5% phenylmethyl sulfonyl fluoride, protease inhibitor cocktail (Hoffmann-La Roche)] and clarification by centrifugation at 14,000 rpm for 15 min a 4°C. Protein samples containing comparable amounts of proteins, estimated by a modified Bradford assay (Bio-Rad), were subjected to western blot and immunocomplexes were detected with the enhanced chemiluminescence kit ECL plus, by Thermo Fisher Scientific (Rockford, IL) using the ChemiDoc (Bio-Rad). Each experiment was done in triplicate.

### Cell proliferation assay

Mesothelioma cells were seeded in 96-multiwell plates and were treated with different doses of indicated two drugs for 72 hours [range dose 10^-7^-10^-12^ M and 0.05-10 μM, for Kp-10 and FTM080, respectively]. Cell proliferation was measured with the MTT assay, as previously described [22]. IC50 were determined by interpolation from the dose-response curves. Results represent the median of three separate experiments, each performed in quadruplicate. Synergism was calculated with ComboSyn software, ComboSyn Inc., Paramus, NK. 07652 USA.

### Invasion and migration assays

The *in vitro* invasive ability of cells was measured by using 24-well Transwell chambers (Corning Life Sciences, MA, USA) according to the manufacturer’s protocol. Briefly, cells were seeded onto the membrane of the upper chamber of the Transwell chambers at a concentration of 5×10^4^/ml in 500 μl of RPMI medium and were treated with the indicated concentrations of drugs for 72 hours [0.001, 0.01, 0.1 nM of Kp-10 and 0.01, 0.1, 1 μM of FTM080]. The medium in the upper chamber was serum-free, the medium at the lower chamber was filled with a normal growth medium containing 10% FBS as a source of chemo-attractants. Cells that passed through the Matrigel coated membrane were stained with Cell Stain Solution containing crystal violet (Chemicon, Millipore, CA, USA) and photographed after 72 hours. Absorbance was measured at 562 nm by an ELISA reader after dissolving of stained cells in 10% acetic acid. Untreated cells served as control and assays were performed in triplicate.

Cell migration was assessed using a commercially available chemotaxis assay [22]. Cells were treated with the same doses used previously and photographed at time 0 (T_0_) and after 72 hours (T_72h_). Assays were performed in triplicate.

### Colony forming assay

Cells were seeded on 6-well tissue culture dishes at 300 cells/well and treated with 0.001, 0.01, 0.1 nM of Kp-10 and 0.01, 0.1, 1 μM of FTM080. All conditions were performed in triplicate and untreated cells were used as control. Cells were maintained for 7 days at which point they were fixed with 4% paraphormaldeid, stained with crystal violet and colonies counted using the GelCount (Oxford Optronix, United Kingdom).

### Zymography assay

Analysis of MMP-2 and MMP-9 activities was assayed by gelatin zymography, as described previously. Briefly, conditioned media from cells cultured in the absence of serum for 48 hours, with the indicated treatments, were collected. Samples (20 μl) were mixed with loading buffer Dye 5X No Redox (1M Tris-HCl pH 6.8, 10% SDS, 50% glycerol, and 0.5% bromphenol blue) and electrophoresed on 8% SDS-polyacrylamide gel containing 0.1% gelatin. Electrophoresis was performed at 100V for 2 hours. Then the gels were washed twice for 10 min at room temperature in zymography washing buffer (2.5% Triton X-100 in double-distilled H_2_O) to remove SDS. Gels were incubated in substrate buffer (40 mmol/L Tris-HCl, 10 mmol/L CaCl_2_, 0.02% NaN_3_ and 1% Triton X-100, pH 8.0) at 37°C for 18 h, stained with Coomassie blue solution (0.125% Coomassie blue R-250, 0.1% amido black, 50% methanol, and 10% acetic acid) for 1 hour and destained with destaining solution (20% methanol, 10% acetic acid, and 70% double-distilled H_2_O).

### Flow cytometry

EpCam surface production was evaluated with a Flow cytometry assay. Cells were washed in staining buffer and then incubated for 30 min at 4°C with mouse anti-human monoclonal antibodies: EpCam-PE or the relative isotype control (MiltenyiBiotec) according to the manufacturer instruction (CD326-EpCAM, MiltenyiBiotec). After this incubation, cells were washes two times in staining buffer and acquired on a FACS ACCURI C6 flow cytometer (BD Biosciences). Analysis was conducted using Accuri C6 software (BD Biosciences).

### *In vivo* metastasis assay

Athymic nude mice (six mice per control group and six mice per treatment group) were inoculated with H2452 mesothelioma cells (20 × 10^5^ cells) via tail vein injection and treated with FTM080 (10 mg/kg p.o.) four days a week, for three weeks. All mice were killed on day 21 [23]. Human DNA in mouse lungs was measured by quantifying Alu sequences through qPCR, as previously described [24].

### Statistical analysis

Results are expressed as means ± s.d. from three or more independent experiments. Differences between groups were assessed by one-way analysis of variance (ANOVA) followed by the Student t-test. For all analyses *P* values represent 2-sided tests of statistical significance effects.
